# Human endoglin as a potential new partner involved in platelet–endothelium interactions

**DOI:** 10.1007/s00018-017-2694-7

**Published:** 2017-10-28

**Authors:** Elisa Rossi, Miguel Pericacho, Christilla Bachelot-Loza, Dominique Pidard, Pascale Gaussem, Sonia Poirault-Chassac, Francisco J. Blanco, Carmen Langa, Consuelo González-Manchón, Jose M. Lopez Novoa, David M. Smadja, Carmelo Bernabeu

**Affiliations:** 1 Centro de Investigaciones Biologicas, Consejo Superior de Investigaciones Científicas (CSIC), Ramiro de Maeztu, 9, 28040 Madrid, Spain; 2 Centro de Investigación Biomédica en Red de Enfermedades Raras (CIBERER), 28040 Madrid, Spain; 3 Paris Descartes University, Sorbonne Paris Cité, Paris, France; 4 Inserm UMR-S1140, Faculté de Pharmacie, Paris, France; 5 Departamento de Fisiología y Farmacología, Unidad de Fisiopatología Renal y Cardiovascular, Universidad de Salamanca, 37007 Salamanca, Spain; 6 Institute for Biomedical Research of Salamanca (IBSAL), Salamanca, Spain; 7 Hematology Department, AP-HP, Hôpital Européen Georges Pompidou, Paris, France

**Keywords:** HHT, Hereditary hemorrhagic telangiectasia, TGF-β, RGD, Preeclampsia, CXCL12

## Abstract

Complex interactions between platelets and activated endothelium occur during the thrombo-inflammatory reaction at sites of vascular injuries and during vascular hemostasis. The endothelial receptor endoglin is involved in inflammation through integrin-mediated leukocyte adhesion and transmigration; and heterozygous mutations in the endoglin gene cause hereditary hemorrhagic telangiectasia type 1. This vascular disease is characterized by a bleeding tendency that is postulated to be a consequence of telangiectasia fragility rather than a platelet defect, since platelets display normal functions in vitro in this condition. Here, we hypothesize that endoglin may act as an adhesion molecule involved in the interaction between endothelial cells and platelets through integrin recognition. We find that the extracellular domain of human endoglin promotes specific platelet adhesion under static conditions and confers resistance of adherent platelets to detachment upon exposure to flow. Also, platelets adhere to confluent endothelial cells in an endoglin-mediated process. Remarkably, Chinese hamster ovary cells ectopically expressing the human αIIbβ3 integrin acquire the capacity to adhere to myoblast transfectants expressing human endoglin, whereas platelets from Glanzmann’s thrombasthenia patients lacking the αIIbβ3 integrin are defective for endoglin-dependent adhesion to endothelial cells. Furthermore, the bleeding time, but not the prothrombin time, is significantly prolonged in endoglin-haplodeficient (*Eng* ^+*/*−^) mice compared to *Eng* ^+*/*+^ animals. These results suggest a new role for endoglin in αIIbβ3 integrin-mediated adhesion of platelets to the endothelium, and may provide a better understanding on the basic cellular mechanisms involved in hemostasis and thrombo-inflammatory events.

**Electronic supplementary material:**

The online version of this article (doi:10.1007/s00018-017-2694-7) contains supplementary material, which is available to authorized users.

## Introduction

Endoglin (Eng; CD105) is a transmembrane glycoprotein, which is expressed by endothelial cells (EC), but not by platelets, and plays a critical physiological role in the cardiovascular system [1–3]. It is an auxiliary receptor for the transforming growth factor β (TGF-β) family of proteins and has been shown to be essential for angiogenesis [4, 5]. The pathophysiological importance of endoglin in vascular biology is clearly established in humans by the linkage between heterozygous mutations in the endoglin gene and the occurrence of the hereditary hemorrhagic telangiectasia (HHT) or Rendu–Osler–Weber syndrome [6]. HHT is considered a vascular disease that presents as epistaxis, mucocutaneous and gastrointestinal telangiectases, and arteriovenous malformations in the pulmonary, cerebral or hepatic circulation [7]. Recurrent nosebleeds, a major presenting sign in HHT patients, can lead to chronic anemia and require blood transfusion [7, 8]. Endoglin was the first gene identified as being involved in HHT [6]. So far, more than 500 different pathogenic mutations in endoglin have been reported, concerning up to 59% of the HHT patient population, thus defined as the HHT1 subtype [9].

Pathogenic mechanisms in HHT are not completely understood, and the bleeding syndrome in patients is postulated to be a consequence of a marked fragility of telangiectases. Nonetheless, recent evidence suggests that HHT1-associated mutations could affect other functions involving blood and vascular cells adhesion [10]. In HHT patients, blood parameters do not show prolonged coagulation time and prothrombin time is normal [11]. However, the mucocutaneous bleeding phenotype could also be due to a defect in primary hemostasis resulting from altered interactions of platelets with the (sub)endothelium. In this context, easy bruisability or post-operative wound healing defects in HHT are suggested by the recommendation of scrupulous dental hygiene and antibiotics prophylaxis prior to dental procedures to avoid the risk of infections [7, 8]. In normal circulation within intact vasculature, platelets barely undergo significant interaction with the endothelial surface. However, at sites of traumatic vascular injury, the subendothelial extracellular matrix (ECM) is exposed, allowing the prompt adhesion of platelets in order to limit hemorrhage, ensure coagulation and promote tissue healing [12–14]. Following de-endothelialization, initial platelet adhesion and spreading on vascular ECM components, such as collagen and von Willebrand factor (VWF), occur through engagement of glycoprotein VI (GPVI) and integrin α2β1, and GPIb-IX-V, respectively [15, 16]. Further stimulation of platelets leads to their aggregation largely through the activation of the αIIbβ3 integrin and its capacity to bind fibrinogen that cross-links adjacent platelets [17]. Actually, the αIIbβ3 integrin can be activated by a variety of soluble molecules, including adenosine diphosphate (ADP), thrombin, as well as inflammatory cytokines such as CXCL12 (SDF-1α) [15, 16, 18, 19]. Moreover, in inflammatory situations, activated EC can become pro-coagulant and pro-thrombogenic and can induce platelet adherence [20–24]. This thromboinflammatory process is not fully understood. As several other integrins, activated αIIbβ3 in platelets interacts with its many plasma or ECM adhesive ligands (fibrinogen, VWF, fibronectin, vitronectin) through recognition of specific sequences in the latter, including the canonical arginine–glycine–aspartic acid (RGD) motif [17, 25]. It is, thus, of interest that the extracellular region of endoglin displays an RGD motif within the ZP-N subdomain [26], which is able to bind, at least, the α5β1 integrin on leukocytes and vascular mural cells upon stimulation of EC by the chemokine CXCL12 [27, 28].

Our study was, thus, aimed to investigate a potential new function for endothelial endoglin based on the hypotheses that: (1) endoglin can be involved in platelet adhesion to the endothelium; (2) the RGD motif present in endoglin could be involved in this cell–cell interaction and platelet integrins α5β1 and/or αIIbβ3, both RGD-binding integrins, be potential counter-receptors for endoglin; and (3) the process of platelet–endothelial cell interactions through an endoglin–integrin axis could be under an inflammatory control via the chemokine CXCL12 and its capacity to reinforce integrin stimulation.

## Materials and methods

### Human blood samples

Citrated blood samples were obtained from healthy donors, HHT1 patients (University Hospital, Salamanca, Spain) and Glanzmann’s thrombasthenia (GT) patients [29], who had not taken any medication for at least 10 days. The ethics committee approved the study and all participants gave written informed consent. Whole blood was centrifuged for 20 min at 100xg to obtain platelet-rich plasma (PRP). PRP was diluted with washing buffer (103 mM NaCl, 5 mM KCl, 2 mM CaCl_2_, 1 mM MgCl_2_, 5 mM glucose and 36 mM citric acid; pH 6.5) and the platelet activation inhibitor PGE1 (2 × 10^−7^ M) and the ADP scavenger apyrase (1 U/mL; Sigma-Aldrich) were added. Then, diluted PRP was centrifuged for 12 min at 1240×*g* to pellet the platelets. This washing step was repeated once, and the washing buffer always contained PGE_1_ and apyrase [30]. Platelets were finally resuspended at 2.5 × 10^8^/mL in Walsh’s buffer (137 mM NaCl, 20 mM PIPES, 5.6 mM dextrose, 1 g/L BSA, 1 mM MgCl_2_, 2.7 mM KCl, 3.3 mM NaH_2_PO_4_, pH 7.4). Prior to any experimental procedure, platelets were typically left at room temperature for 30 min. Under resting conditions, platelets did not show activation, as demonstrated by the lack of staining with an anti-CD62P antibody, a marker of internal storage organelles and their exocytosis during platelet activation. Platelets from GT patients failed to aggregate in response to agonists such as ADP, epinephrine, or collagen [29].

### Cell lines and primary cultures

Human umbilical vein endothelial cells (HUVEC) and human aortic endothelial cells (HAEC) were grown in EBM-2 medium, supplemented by EGM-2 SingleQuots (Lonza). Silencing of endoglin expression in EC, was carried out by nucleofection or lipofection [28]. Generation of stable cell transfectants in L6E9 rat myoblasts, expressing either human L-endoglin or S-endoglin, and Chinese hamster ovary (CHO) cells expressing either human αIIbβ3 or mutant αIIbβ3-P718 has been reported [31, 32].

### Platelet adhesion assays

Plates and coverslips/chamber slides (Millicell EZ slide, Millipore) were coated with 100 µg/mL human fibrinogen (Calbiochem), 1% heat-inactivated BSA, or 7.5 µg/mL soluble endoglin (sEng; extracellular domain Glu26-Gly586, R&D), and then blocked with 0.5% heat-inactivated BSA. Washed platelets were stained with 1 μM calcein (Invitrogen), and aliquots of 10^7^ platelets/250 μL were added to wells of chamber slides. Calcein labeling was shown to neither induce nor alter the activation of platelets. After 5 min at 37 °C, Walsh’s buffer, supplemented with 1 mM CaCl_2_, was added. After centrifugation at 800×*g* for 30 s, plates were incubated for 30 min, washed with PBS and platelet adhesion was quantified by fluorometry using a Varioskan equipment (Thermo Fisher Scientific). For platelet adhesion to EC, calcein-stained platelets were added to HUVEC or HAEC monolayers in the presence/absence of CXCL12 (200 ng/mL), RGD peptide (Arg–Gly–Asp; 1 mM), TRAP6 (20 μM) or CXCR4 inhibitor AMD3100 (1 μM; Sigma-Aldrich), and incubated for 10 min. Individual treatments of EC with CXCL12 or TRAP6 had no impact on endoglin expression levels. For adhesion to protein substrates, calcein-stained platelets were incubated in the absence or presence of CXCL12, mouse monoclonal antibody (mAb) anti-αIIbβ3 integrin (AP-2) [33], or an isotype-matched control IgG1 (MOPC21, Sigma-Aldrich), as indicated. Binding of platelets was quantified by measuring the fluorescence intensity profile using a fluorescence optical microscope (Observer D1, Zeiss) connected to a CCD camera (QIclick FCLR-12, Qimaging, Roper Scientific) and the ImageJ software.

### Flow chamber assays

Microfluidic devices (Fluxion Biosciences or Maastricht Instrumentation) were used to evaluate shear-resistant platelet adhesion [27, 34]. The channels were primed and coated with 0.1% BSA, 7.5 µg/mL sEng or 100 µg/mL fibrinogen. Calcein-labeled platelets were perfused through the channels at 2 dynes/cm^2^, allowed to adhere in the absence of flow for 10 min and then were subjected to 2 dynes/cm^2^ for 2 min. Similar experiments were performed using monolayers of HUVEC, HAEC, parental-L6E9, mock-L6E9, L-endoglin-L6E9, and S-endoglin-L6E9 cells as coating [31]. Under our experimental flow conditions (2 dynes/cm^2^ for 2 min), no changes in endoglin protein levels were observed in endoglin-expressing cells. Platelet adhesion on HAEC was also performed in the presence of human sEng (1 μg/mL), AMD3100, RGD or CXCL12, as indicated above. Real-time platelet adhesion was recorded at 10× magnification using a fluorescence optical microscope (Observer D1, Zeiss) connected with a CCD camera (QIclick FCLR-12, Qimaging, Roper Scientific). Flow rate was adjusted to 1 dyne/cm^2^ (0.2 mL/min) and then increased at 2 dynes/cm^2^ for 2 min.

### Mice and bleeding assays

All procedures were approved by the Committee for the Care and Use of Animals of the University of Salamanca and complied with the current guides of the European Union and the US Department of Health and Human Services for the Care and Use of Laboratory Animals. Endoglin heterozygous (*Eng* ^+*/*−^) mice [35] were backcrossed onto the C57BL/6 background and genotyped as described [36]. The endoglin haploinsufficient mice with C57BL/6 background were selected because they do not show the classical HHT bleeding phenotype [35], which may interfere with the bleeding time assays. To determine the tail bleeding time, animals were anesthetized with isoflurane, their tails were transected at 4 mm from the tip and immediately immersed in PBS at 37 °C. The initial bleeding time, excluding animals with extreme bleeding, was 101.4 ± 66.19 s (*n* = 11) in *Eng* ^+*/*+^ mice and 114.4 ± 78.94 s (*n* = 10) in *Eng* ^+*/*−^ mice, but in some cases rebleeding occurred following initial bleeding arrest. The total bleeding time, taking into account first bleeding plus rebleedings (when occurred), was measured. The percentage of animals with rebleedings that lasted longer than 2 min was also recorded. No rebleeding for 5 min was considered as cessation of bleeding. Prothrombin time and international normalized ratio were quantified using a portable coagulometer (INRatio^®^2, Alere).

### Immunofluorescence flow cytometry

For labeling of cell surface receptors, cells or platelets were incubated for 30 min with monoclonal antibodies anti-GPIbα (Serotec; AK2), anti-αIIb (2BC1), anti-β3 (H1AG11) [37], anti-endoglin (Developmental Studies Hybridoma Bank-DSHB, Iowa; P4A4), or X63 (negative control), followed by incubation with Alexa Fluor 488-anti-mouse IgG (Invitrogen). To detect the binding of soluble endoglin to platelets or CHO transfectants, with or without activation with 10 nM phorbol 12-myristate 13-acetate (PMA), samples were incubated 30–60 min with phycoerythrin-labeled sEng (PE-sEng; R&D/Immunostep) or with unlabeled sEng (R&D). The binding of unlabeled soluble endoglin to platelets was followed by incubation with Alexa Fluor 488-conjugated anti-endoglin antibody (Invitrogen, MHCD10520). All samples were analyzed with a Coulter Epics XL flow cytometer (Beckman Coulter).

### Immunofluorescence microscopy

Samples were incubated with anti-β3 (H1AG11) or anti-tubulin (Sigma-Aldrich) murine monoclonal antibodies and then with Alexa Fluor 488-anti-mouse IgG. For staining of intracellular actin, adherent platelets on coated coverslips were incubated with Alexa Fluor 546 conjugated to phalloidin (Invitrogen). For VWF staining, HAEC monolayers were incubated, first with a primary mouse antibody to VWF (Dako), and then with a Texas Red-labeled secondary antibody to mouse IgG (Vector). Binding of calcein-labeled platelets or CHO transfectants was carried out as described above. Samples were analyzed using the Leica TCS-SP2-AOBS confocal microscope system.

### Western blot analysis

L6E9 myoblasts expressing either human L-endoglin or S-endoglin were lysed on ice-cold lysis buffer (10 mM Tris–HCl pH 8, 150 mM NaCl, 1% NP-40, and a cocktail of protease and phosphatase inhibitors; Roche) and protein concentrations were determined (Bradford, BioRad). Lysate aliquots containing equal amounts of protein were separated by SDS-PAGE and electrotransferred onto PVDF membranes (Millipore). Immunodetection was carried out by probing the membrane with mouse monoclonal antibodies anti-endoglin (DSHB; P3D1) or anti-β-tubulin (Calbiochem), as a loading control, followed by incubation with the corresponding horseradish peroxidase-conjugated secondary antibody. Protein bands were revealed using the SuperSignal chemiluminescent substrate (Pierce).

### Quantification and statistical analysis

All the assays were performed in triplicate and repeated at least twice. Values are expressed as mean ± standard error of the mean (SEM). Data from in vivo experiments are represented as box-plots. Multiple comparison data were analyzed using one-way ANOVA with post hoc Bonferroni and Scheffe tests. Direct group–group comparisons were carried out using independent Student’s *t* tests with prior Levene tests for equality of variances. *p* < 0.01 and *p* < 0.05 were considered statistically significant, and *p* < 0.005 was considered highly statistically significant.

## Results

### Pro-adhesive activity of endoglin on platelets in vitro

**Fig. 1:**
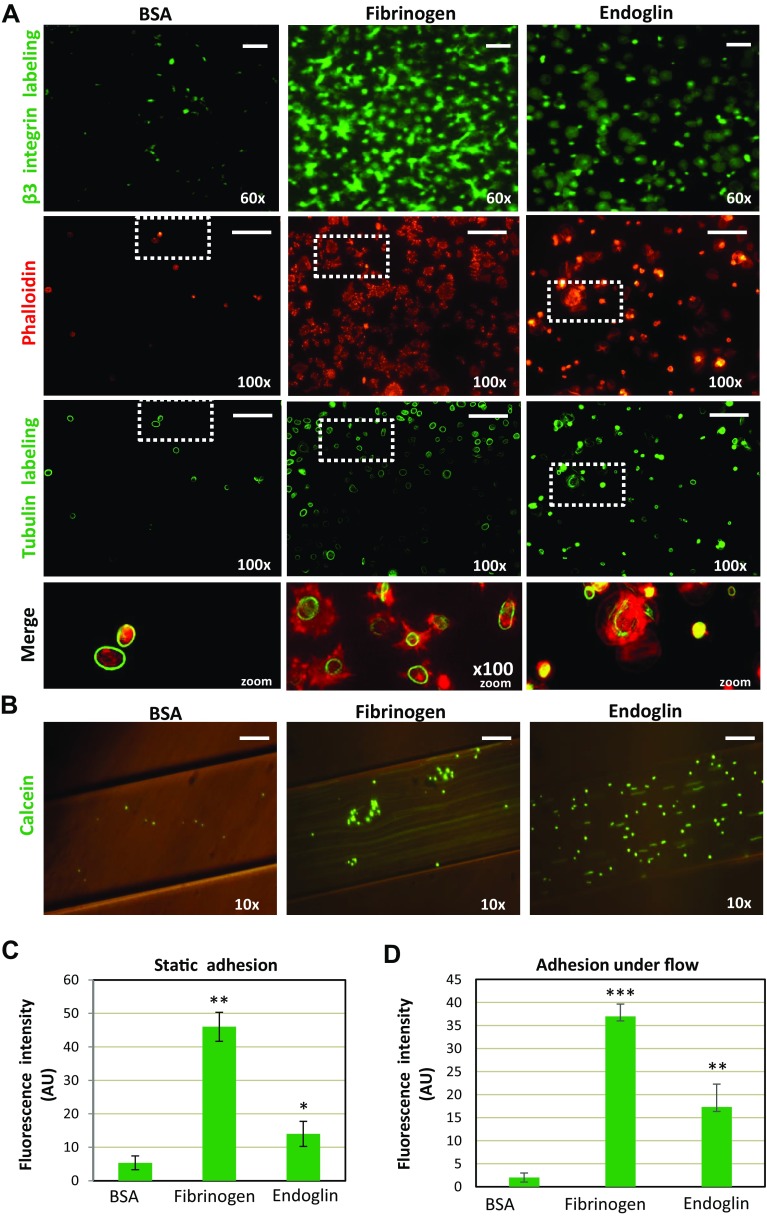
Platelet adhesion on endoglin-coated wells. **a** Platelets resuspended in Walsh’s buffer were incubated for 30 min in endoglin-, fibrinogen- or BSA-coated wells. After washing the plates, platelets were labeled with an anti-β3 integrin subunit antibody (green fluorescence), or with phalloidin (red fluorescence) and anti-tubulin antibody (green fluorescence), as described under “Materials and methods”. The zoom of merged phalloidin and tubulin staining is shown in the lower row. Scale bars, 10 µm. Quantification of platelets labeled with the anti-β3 was performed using ImageJ software and is shown in **c**. Mean values ± SEM of three different preparations of platelets each tested in triplicate are displayed. Significant differences were observed between endoglin or fibrinogen coating and BSA control substrate (**p < 0.005; *p < 0.05). **b** Platelet adhesion under flow was assessed using the microfluidic system of BioFlux, as described under “Materials and methods”. Calcein-labeled platelets were perfused in flow chambers coated with the indicated proteins, allowed to adhere in the absence of flow for 10 min, and then subjected to 2 dynes/cm^2^ for 2 min. Platelets bound to the substrate were visualized by microscopy. Scale bars, 60 µm. Quantification of platelet adhesion was performed using ImageJ software and is shown in **d**. Mean values ± SEM of three different preparations of platelets each tested in triplicate are displayed. Significant differences were observed between endoglin or fibrinogen coating and BSA control substrate (***p < 0.001; **p < 0.005). Of note, due to the different experimental conditions, adhesion values obtained under static (**c**) or flow conditions (**d**) are not comparable. *AU* arbitrary units

To analyze the effects of endoglin on platelet adhesiveness under static conditions, plates were coated with sEng or fibrinogen, a well-known adhesive protein for platelets [38]. Endoglin coating markedly enhanced platelet adhesion with a significant ~ 3-fold increase over nonspecific adhesion to BSA (Fig. 1a, first upper row, c). Adhesion on endoglin was, however, lower than that measured on fibrinogen, which showed a ~ 9-fold increase over BSA. Interestingly, on BSA coating the few bound platelets appear to be non-activated as evidenced by the presence of intact microtubule rings and no rearrangement of the actin cytoskeleton (Fig. 1a; see green stain in third row and red stain in second row, respectively, as well as merge on fourth row). However, on fibrinogen or endoglin coating, adherent platelets were found to be activated, as shown by the presence of widespread microtubule rings, as well as by filopodia, lamellipodia and stress fibers. In the case of fibrinogen, a lack of correlation between tubulin-ring and actin staining was observed, a finding likely due to the fact that fibrinogen induces a strong activation platelets promoting depolymerization of tubulin subunits and loss of tubulin ring-shaped structures. Most of the platelets adherent to fibrinogen under static conditions are spread and sometimes adherent to one another, whereas there were fewer adherent and fully spread platelets on endoglin, suggesting that the mechanism of platelet adherence to fibrinogen or endoglin seems substantially different (see “Discussion”). Of note, on endoglin coating an increased ratio of lamellipodia/filopodia was observed (Supplemental videos #1, 2, 3).

Resistance to flow-induced detachment of platelets adhering on endoglin-coated surfaces was also explored. Platelets were perfused into flow chambers and allowed to adhere to the substrate under static conditions for 10 min and, then, subjected to flow at 2 dynes/cm^2^ for 2 min (Fig. 1b and supplemental videos 4–6). Under this setting, the number of adherent platelets on endoglin or fibrinogen was significantly increased by ~ 7-fold or ~ 14-fold, respectively, as compared to adhesion on BSA (Fig. 1d). Of note, a shear stress of 2 dynes/cm^2^ is within a physiological range, which may vary from 0.4 dynes/cm^2^ (small veins) to 20 dynes/cm^2^ (peak flow in abdominal aorta) [27]. Within this range, low shear stress values are expected to occur in small telangiectases of mucosal membranes, which bleed upon rupture in HHT [7].

### Pro-adhesive activity of endothelial endoglin

**Fig. 2:**
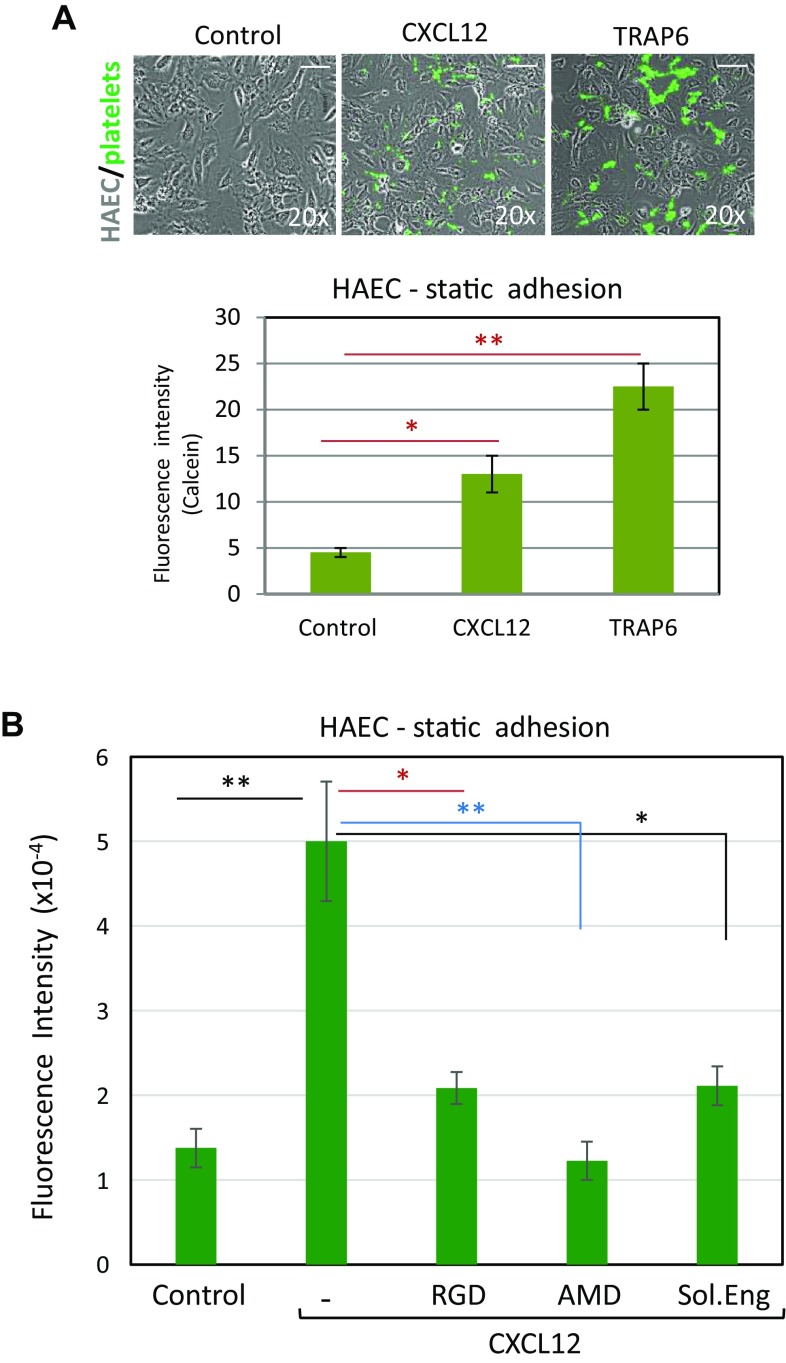
Effect of CXCL12 and TRAP6 on platelet adhesion to endothelial cells. **a** Calcein-labeled platelets were incubated for 10 min on HAEC monolayers in the absence or presence of CXCL12 or thrombin receptor activating peptide 6 (TRAP6) and washed twice with PBS. Adhesion of platelets to EC was visualized by fluorescence microscopy (×20 magnification) (top panels). Scale bars, 40 µm. Quantification of platelet adhesion repeated in quadruplicates with two different platelet preparations and analyzed by ImageJ software is shown in the histogram. Mean values ± SEM are displayed. **p < 0.01; *p < 0.05. **b** Calcein-labeled platelets were incubated for 10 min on HAEC monolayers in the absence (control) or presence of CXCL12, either alone or with the RGD peptide, the CXCR4 inhibitor AMD3100, or the recombinant extracellular part of endoglin (Sol.Eng), as indicated. Platelets were visualized by fluorescence microscopy and quantified (quadruplicates with two different platelet preparations) with ImageJ software. Mean values ± SEM are displayed. **p < 0.005; *p < 0.01

The above results are suggestive of a potential role of endoglin as an adhesive protein for platelets, but do not necessarily imply that endoglin expressed at the EC surface supports platelet adhesion. Thus, platelet adhesion was evaluated on monolayers of cultured EC, which constitutively express endoglin [2, 28]. As indicated in Fig. 2a and supplemental Fig. 1A, B, platelets slightly adhered under static conditions to EC of either venous (HUVEC) or arterial (HAEC) origin. However, this adhesion was markedly enhanced by ~ 5-fold and ~ 3.5-fold on HAEC and HUVEC, respectively, when the integrin-activating chemokine CXCL12 was added to the culture medium. Platelet adhesion to EC was also assayed under shear stress applied to adherent platelets (supplemental Fig. 1C, D, and supplemental videos 7 and 8), showing again that the presence of CXCL12 increased the number of adherent platelets by ~ 2.5- to ~ 3.5-fold over untreated samples. As expected, an increase of platelet adhesion to HAEC was also observed in the presence of the PAR-1 activating peptide TRAP6, a well-known platelet activator [15, 21] (Fig. 2a). Under these conditions, the CXCL12-dependent adhesion of platelets was indeed inhibited by the chemokine receptor (CXCR4) inhibitor AMD3100, but, interestingly, also by the RGD peptide (Fig. 2b). VWF is one RGD-type adhesive protein that can be secreted by activated EC and that can allow adherence of platelets on their surface [21, 22]. Of note, cells exposed to CXCL12 showed a basal labeling of VWF similar to that seen with unstimulated cells, whereas TRAP6 induced a substantial increase of membrane-associated VWF (Supplemental Fig. 2). These results suggest that CXCL12 is not a trigger for VWF secretion under our experimental conditions, and that increased expression of VWF is not involved in the CXCL12-stimulated adhesion of platelets to EC.

**Fig. 3:**
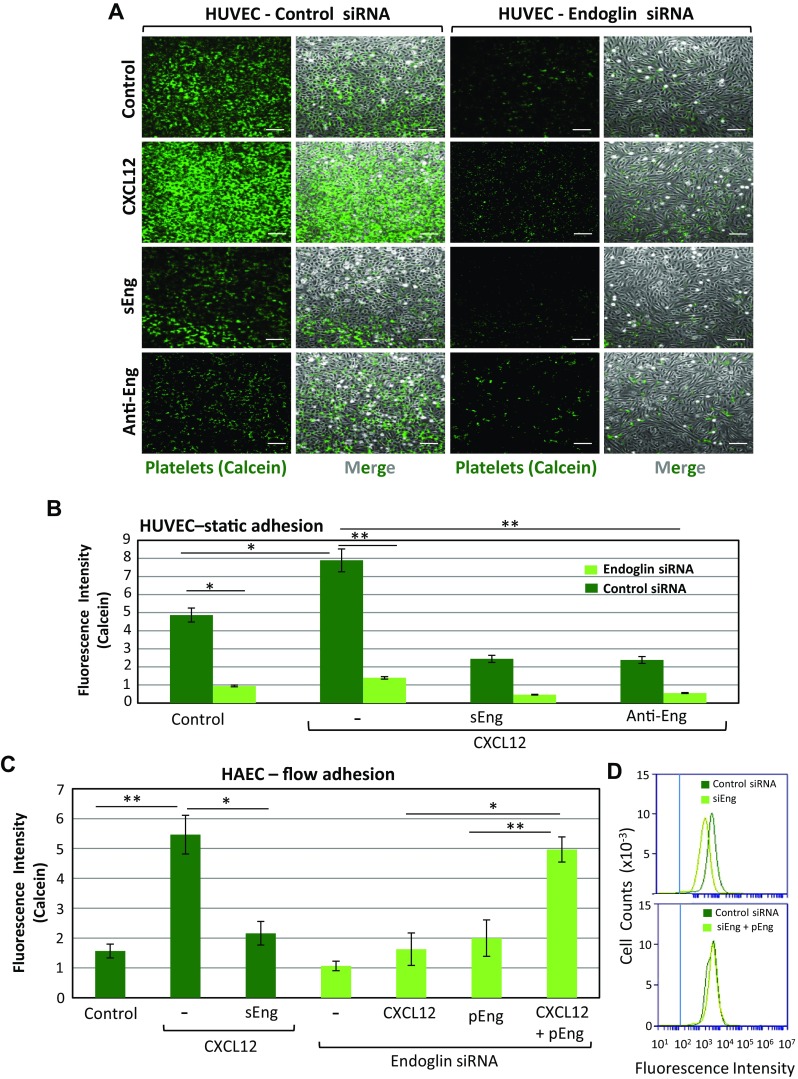
Effect of endoglin silencing on adhesion of platelets to EC. **a** Static adhesion of platelets to HUVEC. Platelets labeled with calcein were incubated for 10 min on HUVEC monolayers, previously treated with a siRNA specific for endoglin or with a scrambled siRNA, as described under “Materials and methods”. Incubation was carried out in the absence or presence of CXCL12 (100 ng/mL), sEng (50 ng/mL), or the anti-endoglin mAb P4A4. After washing twice with PBS, adhesion under static conditions was visualized by fluorescence microscopy (×20 magnification). Scale bars, 40 µm. **b** Quantification of platelet adhesion shown in **a** repeated in triplicates with two different platelet preparations and analyzed using ImageJ software. Mean values ± SEM are displayed. **p < 0.005; *p < 0.05. **c** Platelet adhesion under flow was assessed using a Maastricht Instrumentation equipment. Calcein-labeled platelets were perfused in flow chambers coated with HAEC, previously untreated or treated with siRNA specific for endoglin, in the presence of CXCL12 or sEng, as indicated. Rescue experiments of endoglin expression were performed by nucleofection of HAEC with the endoglin expression vector pCEXV-EndoL (pEng). Platelets were allowed to adhere in the absence of flow for 10 min, and then subjected to 2 dynes/cm^2^ for 2 min. Quantification of platelet adhesion (triplicates with two different platelet preparations), was performed using ImageJ software. Mean values ± SEM are displayed. **p < 0.005; *p < 0.05. **d** HAEC were nucleofected with siRNA specific for endoglin (siEng) or with a scrambled siRNA (control siRNA), as indicated. Rescue experiments of endoglin expression were performed by nucleofection of HAEC with the endoglin expression vector pCEXV-EndoL (pEng). The expression levels of endoglin were determined by immunofluorescence flow cytometry using specific antibodies prior to adhesion experiments shown in **c**. The vertical blue line indicates the fluorescence intensity of HAEC stained with an irrelevant isotype-matched control antibody

Using flow cytometry, we previously reported that HAEC and HUVEC express high and similar levels of endoglin, whereas endoglin expression was markedly reduced in EC nucleofected with endoglin-specific siRNA, but not with a scrambled, negative control siRNA [28]. Moreover, endoglin-silenced EC could return to normal levels of endoglin expression upon rescue with the endoglin expression vector pCEXV-EndoL [28]. Thus, a role for endoglin in the adherence of platelets to EC as described above could be inferred from the following observations. Firstly, endoglin silencing in HUVEC or addition to the medium of either sEng, as a competitor for membrane-bound endothelial endoglin, or the blocking anti-endoglin mAb P4A4, all markedly and significantly (*p* < 0.001) inhibited the adherence of platelets to HAEC or HUVEC in the presence of CXCL12 (Figs. 2b, 3a, b). As measured under static conditions, a reduction of ~ 70–80% in the number of adherent platelets was found when membrane-bound endothelial endoglin was suppressed/neutralized as compared to untreated HUVEC (Fig. 3b). Interestingly, silencing endoglin expression also significantly reduced by ~ 80% the adherence of platelets to HUVEC in the absence of CXCL12 stimulation. In addition, upon CXCL12 stimulation, combining endoglin silencing with competition or blockade of residually expressed endothelial endoglin with sEng or the anti-endoglin mAb reduced the number of adhering platelets on HUVEC to less than 10% of control values (Fig. 3b). This residual endoglin-independent binding of platelets to EC probably involves other adhesion molecules, including VWF, which can be spontaneously released during culture of EC (Supplemental Fig. 2), and various platelet membrane receptors [21, 22]. The role of endoglin in platelet–EC interaction was confirmed when looking at the resistance of adherent platelets to detachment upon exposure to flow (Fig. 3c and supplemental videos #9–12). In these experiments, we used either endoglin-silenced HAEC or cells previously exposed to sEng that acts as an endoglin competitor. Platelets remaining adherent to HAEC under flow in the presence of CXCL12 were significantly (*p* < 0.005) reduced by ~ 70% upon silencing of endoglin expression, and by approximately 60% in the presence of sEng (Fig. 3c). In the absence of CXCL12, silencing endoglin expression also reduced by ~ 40% (*p* < 0.005) the number of platelets that adhered to HAEC. Restoring endoglin expression in endoglin-silenced HAEC using an endoglin expression vector allowed the recovery of normal values of platelet adhesion to either unstimulated or CXCL12-stimulated samples (pEng, Fig. 3c, d). Of note, individual treatments of HAEC with CXCL12 had no impact on endoglin expression levels (data not shown).

**Fig. 4:**
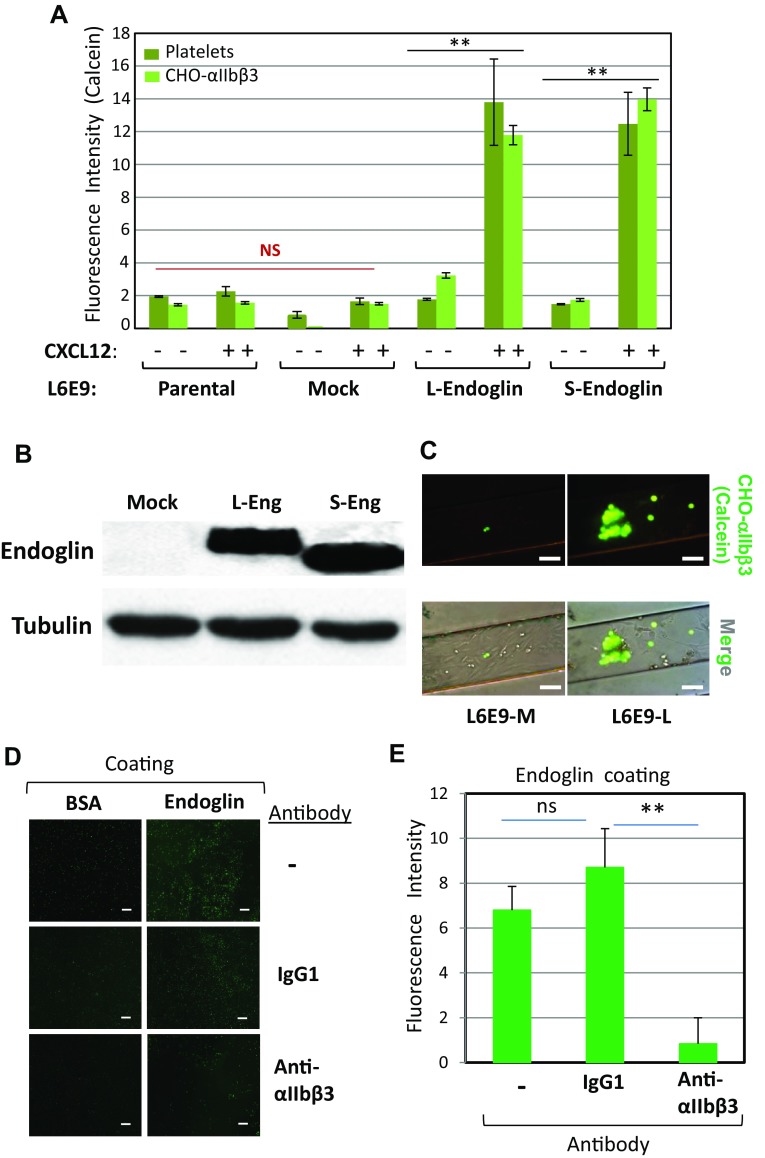
Involvement of αIIbβ3 integrin in endoglin-mediated cell adhesion. **a** Adhesion of platelets and CHO-αIIbβ3 cells to endoglin-expressing cells was measured under flow. Calcein-labeled platelets or CHO-αIIbβ3 cells were perfused in flow chambers previously coated with parental (L6E9-P), mock-transfected (L6E9-M), L-endoglin-transfected (L6E9-L) or S-endoglin-transfected (L6E9-S) cells in the absence or presence of CXCL12, allowed to adhere in the absence of flow for 10 min, and then subjected to 2 dynes/cm^2^ for 2 min. Quantification of adhesion, repeated in triplicates with different platelets (dark green) or CHO cells (light green) preparations, was performed by ImageJ software. Mean values ± SEM are displayed. **p < 0.005; *NS* not significant. Parallel experiments using calcein-labeled CHO wild-type cells did not show any significant adhesion to endoglin-expressing L6E9 transfectants (fluorescence levels < 2; data not shown). **b** Western blot analysis of endoglin in L6E9 mock, L-endoglin (L-Eng) and S-endoglin (S-Eng) transfectants, using tubulin as a loading control. **c** Representative experiment of adhesion of calcein-labeled CHO-αIIbβ3 cells to flow chambers coated with mock-transfected cells (L6E9-M) or L-endoglin-transfected cells (L6E9-L), performed as described in **a** and visualized by fluorescence microscopy (×10 magnification). Scale bars, 20 µm. **d**, **e** Platelet adhesion to endoglin is inhibited by anti-β3 antibodies. Plates coated with BSA or endoglin were incubated with calcein-labeled platelets for 15 min in the absence (−) or in the presence of an anti-αIIbβ3 integrin mAb or an isotype-matched control IgG1. After washing with PBS, plates were visualized by fluorescence microscopy (×10 magnification). Scale bars, 40 µm (**d**). Quantification of adhesion was performed using ImageJ software (**e**). Background adhesion values of BSA-coated wells were subtracted from those of endoglin-coated wells. Mean values ± SEM are displayed. **p < 0.005

Next, we used the rat myoblast cell line L6E9-P as another cellular model, since these cells do not express endoglin, but can be stably transfected with the long (L6E9-L) or the short (L6E9-S) form of human endoglin [31]. These cell transfectants may serve to assess not only the role of the extracellular domain, shared by both isoforms, but also the putative role of the cytoplasmic domain, which markedly differs in length, amino acid composition and phosphorylation status between L-endoglin and S-endoglin [2]. We, thus, compared the adhesion of platelets to monolayers of parental, untransfected (L6E9-P) or mock-transfected (L6E9-M) cells, with adhesion to cells expressing either one of the two endoglin isoforms (L6E9-L or L6E9-S). Stimulation with CXCL12 did not allow noticeable platelet adhesion under flow to either L6E9-P or L6E9-M cells. However, and similar to results obtained with EC, it induced a significant (*p* < 0.005) and marked platelet adhesion to L6E9-L and L6E9-S cells, with a seven- to eightfold increase over baseline values (Fig. 4a, see dark green bars). L- and S-endoglin in transfected L6E9 cells could be detected to similar levels by Western blot analysis, while showing a slight molecular mass difference between both isoforms (Fig. 4b), as reported [31].

### Platelet αIIbβ3 integrin is involved in endoglin-mediated adhesion to endothelial cells

In search for counter-receptor(s) on platelets that could interact with endothelial endoglin, and given that we have previously demonstrated that the endoglin RGD motif is involved in integrin-mediated cell adhesion to EC [27, 28], we focused our attention on the αIIbβ3 integrin, a major RGD-dependent adhesive receptor in platelets [17, 25]. We performed flow chamber adhesion assays using our several L6E9 cell subtypes as adherent cell monolayers, and CHO cells either negative for human αIIbβ3 (wild-type CHO) or transfected to express human αIIbβ3 (CHO-αIIbβ3), as flowing cells. Under our experimental conditions, wild-type parental CHO cells never displayed adhesion to any of the L6E9 cell monolayers (data not shown). However, expression of human endoglin in L6E9-L or L6E9-S promoted adhesion of CHO-αIIbβ3 cells in the presence of CXCL12 (Fig. 4a–c). To confirm that endoglin is able to bind αIIbβ3 integrin, we performed binding assays using confocal microscopy and flow cytometry analysis of CHO cell lines exposed to phycoerythrin (PE)-labeled sEng (Supplemental Fig. 3). The extracellular domain of endoglin was able to bind CHO-αIIbβ3 cells upon their stimulation with PMA, as well as CHO cells expressing a constitutively activated αIIbβ3 integrin (CHO-αIIbβ3-P718) [32], but not αIIbβ3-negative CHO cells (Supplemental Fig. 3A). Flow cytometry analysis also revealed PE-sEng binding in PMA-stimulated CHO-αIIbβ3 cells, and in unstimulated CHO-αIIbβ3-P718 cells, but not in parental CHO (Supplemental Fig. 3B). Fluorescence was extensively reduced in the presence of a tenfold molar excess of unlabeled sEng, supporting binding specificity. Moreover, binding of unlabeled sEng to PMA-stimulated platelets was evidenced using a fluorochrome-conjugated antibody against endoglin (Supplemental Fig. 3C).

Additional evidence for the interaction between the platelet αIIbβ3 integrin and endoglin was obtained from experiments of platelet adhesion to endoglin-coated plates (Fig. 4d, e). In these assays, we used the antibody AP-2 to αIIbβ3 integrin, which has been reported to block fibrinogen-dependent platelet aggregation [33], as well as platelet adhesion to immobilized substrates such as fibrinogen [39]. Thus, we found that binding of platelets to endoglin was clearly inhibited by addition of the blocking antibody AP-2 to the αIIbβ3 integrin, as compared to a control mouse IgG1 (Fig. 4e).

**Fig. 5:**
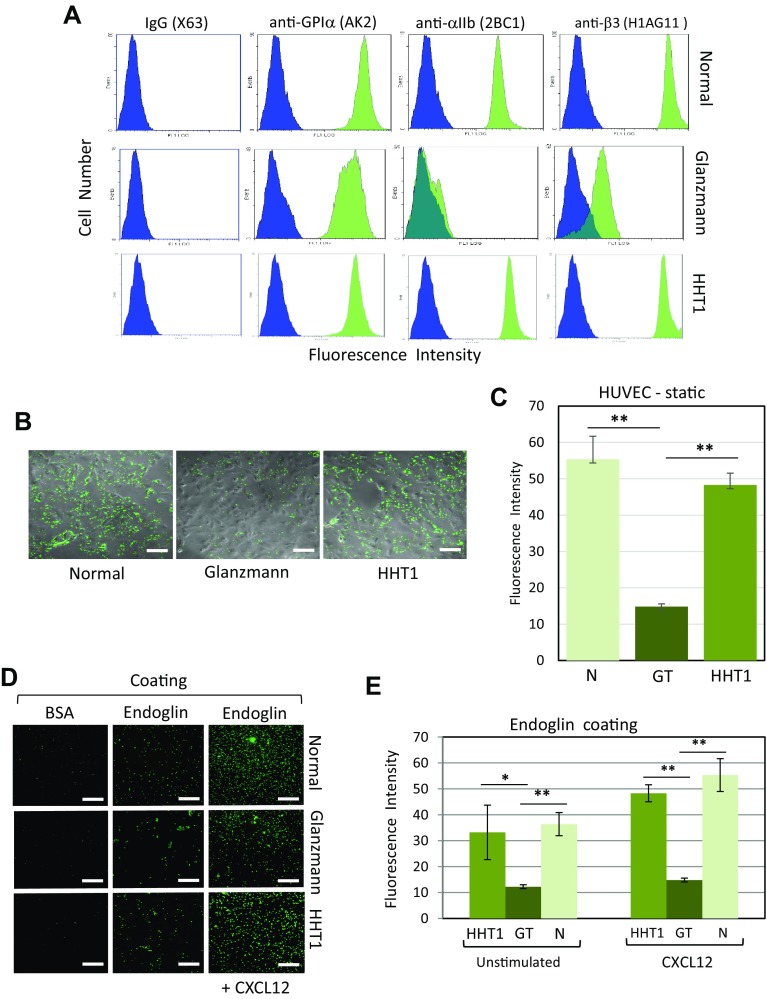
Adhesion of platelets from patients with GT or HHT1 diseases. **a** Integrin expression in platelets from normal subjects, and GT and HHT1 patients was analyzed by flow cytometry using anti-GPIα (AK2), anti-αIIb (2BC1), and anti-β3 (H1AG11) mAbs. As a negative control, non-specific IgG (X63) was used. Representative histograms are shown and illustrate a deficient αIIbβ3 integrin expression in GT platelets, whereas the GPI-V-IX complex is expressed to normal levels. **b**, **c** Adhesion of platelets from normal subjects, and GT and HHT1 patients to HUVEC monolayers. Calcein-labeled platelets were incubated for 15 min on HUVEC monolayers in the presence of CXCL12. After washing three times with PBS, representative samples were visualized by fluorescence microscopy (×20 magnification). Scale bars, 40 µm (**b**). Each platelet preparation from patients or control subjects was analyzed twice (in consecutive days) using three replicates each time (normal subjects, *n* = 10; GT, *n* = 4; HHT1, *n* = 6). Quantification of adhesion was performed using ImageJ software (**c**). Mean values ± SEM are displayed. **p < 0.005. **d**, **e** Adhesion of platelets from normal subjects, and GT and HHT1 patients to endoglin substrate. Plates coated with BSA, endoglin, or endoglin plus CXCL12 were incubated with calcein-labeled platelets for 15 min. After washing with PBS, plates were visualized by fluorescence microscopy (×20 magnification). Scale bars, 40 µm **(d)**. Each platelet preparation from patients or control subjects was analyzed twice (in consecutive days) using three replicates each time (normal subjects, *n* = 10; GT, *n* = 4; HHT1, *n* = 6). Quantification of adhesion was performed using the Varioskan equipment (**e**). Background adhesion values of BSA-coated wells were subtracted from those of endoglin-coated wells. Not significant differences were observed between HHT1 and normal platelets. Mean values ± SEM are displayed. **p < 0.005; *p < 0.01

Finally, a major role of the platelet αIIbβ3 integrin in the process of endoglin-dependent platelet adhesion to EC was supported by the use of platelets from two Glanzmann thrombasthenic (GT) patients [29]. Flow cytometry analysis of the major membrane adhesive receptor subunits GPIα, αIIb and β3 indicated normal levels of GPIα in GT as compared to control normal platelets, while GT platelets showed a drastic reduction in the expression levels of both αIIb and β3 subunits (Fig. 5a). In static adhesion assays using HUVEC monolayers, GT platelets in the presence of CXCL12 showed a significant ~ 70% reduction of adherence as compared to normal platelets (Fig. 5b, c). In the same assay, platelets from three different HHT1 patients expressing normal levels of αIIbβ3 integrin (Fig. 5a) showed a normal level of adhesion to HUVEC (Fig. 5b, c). In addition, binding of GT platelets to endoglin-coated plates was markedly decreased as compared to platelets from normal subjects or HHT1 patients, this difference being enhanced in the presence of CXCL12 (Fig. 5d, e). Together, these results suggest that endoglin can interact with αIIbβ3 integrin present on platelets.

### Endothelial endoglin-deficient mice exhibit an increased bleeding time

**Fig. 6:**
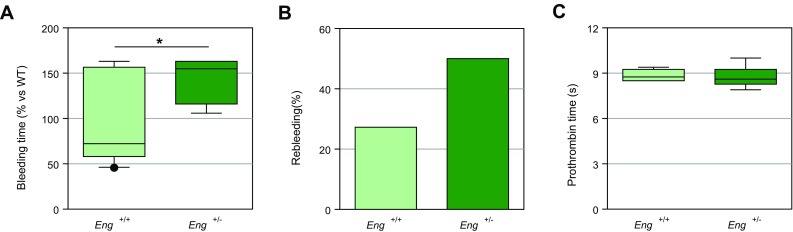
Bleeding and prothrombin times in heterozygous endoglin-deficient mice. **a** Total bleeding time (initial bleeding plus rebleedings) in *Eng* ^+*/*−^ mice (*n* = 10) is significantly longer (*p < 0.05) than that of *Eng* ^+*/*+^ animals (*n* = 11). The mean bleeding time of *Eng* ^+*/*+^ mice was taken as a reference and each measurement was expressed as percentage of this value. **b** Rebleeding was assessed following initial bleeding arrest. A total of 11 *Eng* ^+*/*+^ and 10 *Eng* ^+*/*−^ mice were used and the percentage of animals showing rebleeding longer than 2 min is shown. **c** Prothrombin time in *Eng* ^+*/*−^ mice (*n* = 10) is not significantly different from control animals (*n* = 11). *s* seconds. Graphs in **a**, **c** are displayed as box-plots including median values

Endoglin heterozygous mice (*Eng* ^+*/*−^) represent a model of HHT1 [35]. Endoglin haploinsufficiency in these mice is confirmed by a reduction of ~ 35–65% in the amount of endoglin protein in highly vascularized tissues (lungs and kidneys) and ~ 50% in cultured aortic EC [40, 41]. We observed that *Eng* ^+/−^ mice in C57BL/6 background had significantly prolonged bleeding times as compared to wild-type *Eng* ^+*/*+^ animals (initial bleeding plus rebleedings) (Fig. 6a), as well as an increased number of animals with long rebleedings [5/10 *Eng* ^+*/*−^ mice (50%) vs 3/11 *Eng* ^+*/*+^ mice (27.27%)] (Fig. 6b). However, no differences in prothrombin time between *Eng* ^+/−^ and wild-type mice were found (Fig. 6c), in agreement with published data from HHT1 patients [11]. Because *Eng* ^+*/*−^ C57BL/6 mice do not show evident vascular anomalies [35, 36, 41], their increased bleeding time cannot be explained by the presence of HHT-like vascular lesions, but likely by their endoglin haploinsufficiency in the endothelium. Taken together, these results are compatible with the potential involvement of endothelial endoglin in primary hemostasis.

## Discussion

Intricate interactions between circulating platelets and an activated microvascular endothelium are at the basis of the thrombo-inflammatory reaction seen at sites of vascular injuries accompanying many pathophysiological situations [13, 20], and are likely an early step during vascular hemostasis [23, 24]. However, these interactions still remain to be fully characterized at the molecular level. Focal interaction of (activated) platelets with an (activated) endothelial surface was initially proposed to be mediated by activation of the coagulation, and/or the dampening of the anticoagulation, anti-platelet, and fibrinolytic cascades on EC, thus allowing the formation of an endothelium-associated fibrin network able to entrap platelets [42–44]. However, platelets and EC may also show direct interactions through engagement of adhesion receptors, counter-receptors, and ligands, that allow platelets to first roll, then adhere and possibly aggregate on the endothelial surface [45–47]. It is commonly accepted that subsequent to rolling, firm adhesion of platelets to EC and accompanying platelet activation occurs mainly via the engagement of integrins on both sides, the αVβ3 on EC and the αIIbβ3 on platelets that can bind several RGD-type adhesive proteins acting as bridging molecules between the two cell types [21–23, 48].

**Fig. 7:**
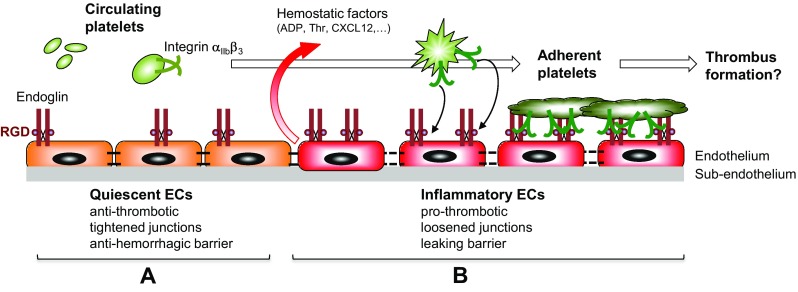
Role of endothelial endoglin in platelet adhesion to the thrombo-inflammatory endothelium. The schematic diagram focuses on a hypothetical model for endoglin-mediated adhesion of platelets to the (micro)vascular endothelium. **a** Under normal conditions, circulating platelets do not adhere to a quiescent endothelium, which displays anti-thrombotic properties, tightened junctions and acts as an anti-hemorrhagic barrier. **b** On inflammatory stimulation, the endothelium shifts to a pro-thrombotic state, shows loosened junctions and behaves as a leaking barrier. Under this setting, EC release different soluble factors, including adenosine 5′-diphosphate (ADP), thrombin (Thr) and the chemokine CXCL12, leading to activation of platelet integrin αIIbβ3. In turn, activated integrin αIIbβ3 can bind to endoglin on EC, allowing adhesion of platelets to the endothelium. The presence of the juxtamembrane RGD motif within endoglin is indicated as a brown sphere. The involvement of other adhesion receptors on both cell types, such as those described in the text, has been omitted for simplicity

A potential new partner in platelet–EC interactions is now emerging, as our data support the involvement of endothelial endoglin in platelet adhesion to endothelium through a process that clearly implicates platelet αIIbβ3, the quantitatively predominant integrin family member in platelets [49, 50], as a counter-receptor (Fig. 7). Thus, platelets could specifically adhere to purified, immobilized endoglin, and a drastic reduction of platelet adhesion to EC was observed when endoglin was silenced with siRNA or in the presence of either sEng, the extracellular part of endoglin containing the RGD motif, or the anti-endoglin P4A4 antibody. Moreover, the effect of endoglin silencing was rescued with the ectopic expression of endoglin. Finally, the specific involvement of endoglin in platelet adhesive function is also supported by experiments using myoblast transfectants overexpressing cell surface human endoglin. Recent studies have shown that endoglin binds to integrins on leukocytes and vascular mural cells via its RGD motif [27, 28]. Integrin αIIbβ3 is a receptor for fibrinogen, VWF, fibronectin and vitronectin, involving their RGD motifs and contributes to platelet activation. Using several cell transfectants expressing either αIIbβ3 integrin or endoglin, we now demonstrate the specific interaction between αIIbβ3 and endoglin under shear stress conditions. Also, the specific binding of sEng to CHO cells expressing human αIIbβ3 is shown in the present work, whereas platelets from GT patients, which exhibit a deficient expression of αIIbβ3 integrin, display a strong reduction of adhesion to endoglin-coated plates, as well as to endoglin-expressing EC, compared to control platelets. The low level of residual adhesion shown by GT platelets (see Fig. 5) could be due to other adhesion molecules and cell receptor pairs, such as secreted VWF, the GPIb-V-IX complex, or αvβ3 or α5β1 integrins [21, 22, 48]. In this regard, we have already demonstrated that α5β1 integrin is involved in endoglin-mediated adhesion [27]. Although the RGD-dependent integrin α5β1 is present on platelets and supports their adhesion to fibronectin, it is expressed at much lower levels than αIIbβ3 [49, 50]. Interestingly, adhesion of normal platelets to an endoglin-coated surface led to the formation of lamellipodia, as well as to a widespread of the microtubule ring, both processes being the consequence of the integrin-mediated activation of platelets [51, 52]. However, we noted a differential behavior of platelet adherence to fibrinogen or endoglin that is likely related to the fact that fibrinogen is an hexamer with at least four integrin-binding motifs (two at each end, one RGD and one dodecapeptide) that would allow full outside–in signaling and clustering of integrins, whereas endoglin is a dimeric protein with only two RGD integrin-binding motifs. These differences in adherence may reflect the existence of distinct binding affinities and specificities of these ligands to αIIbβ3 and other platelet integrins.

On circulating platelets in vivo, the αIIbβ3 integrin is in an inactive form, thus preventing spontaneous platelet aggregation in the circulation. However, upon platelet activation at sites of vascular injury, αIIbβ3 is readily activated for ligand binding, enabling the formation of hemostatic plugs [38, 50]. This is in line with our results showing that: (1) treatment of platelets with the PAR-1-activating peptide TRAP6 or with CXCL12 stimulates platelet adhesion to EC, while treatment of platelets with CXCL12 enhances the binding of platelet αIIbβ3 to endoglin in static and flow conditions; and (2) constitutively activated αIIbβ3 integrin (mutant P718) and wild-type αIIbβ3 activated with phorbol esters in CHO cells show an enhanced binding to sEng. Several studies have demonstrated the CXCL12-induced activation of platelets [18, 53–55], including their increased adherence to EC under flow conditions [56]. Moreover, the involvement of the CXCL12/CXCR4/CXCR7 signaling axis in the activation of platelet integrins is in agreement with: (1) the presence of the CXCL12 receptors CXCR4 and CXCR7 in the platelet membrane [19, 57]; (2) the recent observations that CXCL12 is an autocrine activator of platelets [19, 58]; and (3) the depletion of CXCR4 in mice results in smaller thrombi [59]. Thus, once activated under an inflammatory environment, platelet integrins may bind to endothelial endoglin (Fig. 7). Supporting this view, endoglin has been shown to be upregulated in vivo in several inflammatory settings, including wound healing, atherosclerosis and chronic kidney disease [60–62]. Interestingly, high plasma levels of a soluble form of endoglin are found in patients with preeclampsia, a leading cause of maternal and prenatal morbidity associated with systemic hypertension, chronic inflammation and thrombocytopenia [63–66]. This soluble form of endoglin is thought to be released from membrane-bound endoglin upon the proteolytic action of the metalloprotease MMP-14 [67–69]. Because soluble endoglin has been postulated to have a pathogenic role in preeclampsia [63, 68, 70] and retains its capacity to bind integrins ([27, 28]; this work), it will be interesting to explore whether soluble endoglin has an impact on platelet function in this disorder.

Together, the above data suggest a close physical and functional association between endoglin on EC, and the αIIbβ3 integrin on platelets, that could be central in the interactions between the two cell types in a thrombo-inflammatory context (Fig. 7). Platelet–EC physical interactions share many features with leukocytes interacting with an inflammatory endothelium in the process of extravasation [46, 47, 71, 72]. As mentioned above, platelets first tether and roll on the endothelium, then arrest, firmly adhere, spread, and finally get fully activated [46, 47]. After the initial tethering and rolling steps [21, 23, 24, 73], arrest and firm adhesion, including spreading of platelets to EC appear to essentially implicate the αIIbβ3 integrin on the platelet side, while several counter-receptors have been identified on activated EC. The latter include the αVβ3 integrin, with bridging of the two integrins by their shared, RGD-type adhesive proteins fibrinogen and/or VWF, as well as intercellular adhesion molecule-1 (ICAM-1) through bridging by fibrinogen [22, 48, 74]. Remarkably, however, the combination of blocking antibodies targeting simultaneously the αVβ3 integrin and ICAM-1 on EC appears to reduce the platelet–EC interaction by no more than 50% [22], suggesting that other endothelial receptor(s) could be involved in the final step of firm platelet adherence and spreading. Based on our present observation, endoglin is likely to be one such receptor, and it is of note that, under our experimental conditions, blocking endothelial endoglin expression almost completely abrogated the firm adhesion of platelets to EC and their resistance to detachment under flow.

Although endoglin is a TGF-β co-receptor, in this work we have not explored whether endoglin-dependent adhesion to integrins is mediated by TGF-β signaling. Nonetheless, several lines of evidence suggest that these are two independent processes. First, integrins bind to the RGD-containing zona pellucida domain (ZPD) [27, 28], whereas the physiological ligand BMP9, a member of the TGF-β family, binds to the orphan domain (OD); and both ZPD and OD are in separate regions of the extracellular part of endoglin [75]. Second, cells overexpressing L-endoglin or S-endoglin show a similar level of integrin-dependent binding (Fig. 4), in spite of the fact that these isoforms contribute to opposite TGF-β signaling pathways mediated by the type I receptors ALK1 or ALK5, respectively [76]. Finally, when coated to the substrate, purified endoglin is able to mediate binding to integrins, thus ruling out the potential involvement of endoglin-dependent intracellular signaling in these assays (Figs. 1, 4). Future studies may be needed to further assess the potential crosstalk between the TGF-β signaling pathway and endoglin-dependent binding to integrins.

The early formation of a blood cell plug to seal a disrupted endothelial barrier is a repair mechanism whose alteration could play a critical role in vascular pathologies such as HHT1. Although it is assumed that HHT1 is a vascular disease due to the fragility of the vessels, the involvement of an impaired mechanism of hemostasis when forming the blood cell plug cannot be excluded. As we provide evidence that endoglin plays a role in platelet adhesion on endothelium by interacting with integrins, an impaired adhesive function due to endoglin haploinsufficiency in EC might be involved in the associated bleeding in HHT1 patients. This is in line with the finding that *Eng* ^+*/*−^ mice, a model for HHT1, have a prolonged bleeding time compared to controls (*Eng* ^+*/*+^). Moreover, in HHT1 patients [11] and *Eng* ^+*/*−^ mice, the prothrombin time is similar to controls, suggesting a normal platelet-dependent extrinsic pathway of coagulation. Although many vascular disorders may lead to hemorrhage or thrombosis, it is often difficult to discern between a primary vascular defect/damage and a defect that has been induced by platelet activation/dysfunction or procoagulant abnormalities. Therefore, by deciphering the molecular mechanisms involved in HHT1-associated chronic bleedings, we may contribute to optimize current treatments and find novel therapeutic targets for other bleeding and/or thrombotic disorders.

## Electronic supplementary material

Below is the link to the electronic supplementary material.

Supplementary material 1 (AVI 1212 kb)

Supplementary material 2 (AVI 2697 kb)

Supplementary material 3 (AVI 1531 kb)

Supplementary material 4 (MOV 2593 kb)

Supplementary material 5 (MOV 4202 kb)

Supplementary material 6 (MOV 3133 kb)

Supplementary material 7 (MOV 3004 kb)

Supplementary material 8 (MOV 3819 kb)

Supplementary material 9 (AVI 2949 kb)

Supplementary material 10 (MP4 1916 kb)

Supplementary material 11 (MP4 1920 kb)

Supplementary material 12 (MP4 1928 kb)

Supplementary material 13 (PDF 1344 kb)
