# Supplementary material for: Human endoglin as a potential new partner involved in platelet–endothelium interactions
Source: Cell Mol Life Sci. 2017 Oct 28;75(7):1269–84. doi: 10.1007/s00018-017-2694-7 (PMC5843676; doi:10.1007/s00018-017-2694-7)
Supplement: Supplementary file 13 — Supplementary material 13 (PDF 1344 kb) [file 18_2017_2694_MOESM13_ESM.pdf]

SUPPLEMENTAL MATERIAL

LEGENDS TO SUPPLEMENTAL FIGURES AND VIDEOS

Supplemental Figure 1. **Platelet adhesion on endothelial cells**. **A.** Calcein-labeled platelets were incubated for 10 min on HAEC or HUVEC monolayers in the absence or presence of CXCL12, and washed twice with PBS. Adhesion of platelets to EC under static conditions was visualized by confocal microscopy (10x magnification). **B.** Quantification of platelet adhesion shown in **A** repeated in quadruplicates with two different platelet preparations and analyzed by Image J software. Mean values ±SEM are displayed. **C.** Platelet adhesion under flow was assessed using the microfluidic system of BioFlux, as described under Methods. Calcein-labeled platelets were perfused in flow chambers coated with HAEC or HUVEC in the absence or presence of CXCL12, allowed to adhere in the absence of flow for 10 min, and then subjected to 2 dynes/cm^2 for 2 min. Platelets bound to the substrate were visualized by microscopy and BioFlux 1000z system (10x magnification). **D**. Quantification of platelet adhesion shown in **C** repeated in quadruplicates with two different platelet preparations and analyzed by Image J software. Mean values ± SEM are displayed. ***p<0.001; **p<0.005; *p<0.05.

Supplemental Figure 2. Staining of VWF in endothelial cells. A. Monolayers of HAEC were treated or not with CXCL12 (100 ng/ml) or TRAP6 (100 µM) for 10 min, as indicated. Cells were permeabilized or not (right and left columns, respectively) with 0.1% Triton in PBS for 4 min at room temperature and samples were stained with Texas Red-labeled antibody to VWF, Oregon Green-labeled phalloidin (for the actin cytoskeleton) and Hoechst (for nuclei). After washing with PBS, staining of VWF (red), actin (green) and nuclei (blue) was visualized by fluorescence microscopy (40x magnification) using the Leica TCS-SP2-AOBS confocal microscope. Scale bars, 10 µm. Non-activated and non-permeabilized HAEC cultures show a labeling of VWF that is partly extracellular, with a fibril-like appearance corresponding to VWF associated to the endothelial ECM (see white arrows), while some dot-like labeling represents membrane-associated VWF extruded from secreted Weibel-Palade bodies (WPB). Upon permeabilization, endothelial cells showed rather similar VWF staining in control and CXCL12- or TRAP6-treated samples. In non-permeabilized HAEC exposed to TRAP6, patches of membrane-associated VWF were much more intense,

indicating that the basal extrusion of WPB had been stimulated. By contrast, cells exposed to CXCL12 showed a basal labeling of VWF very similar to that seen with unstimulated cells. **B.** Quantification of the VWF-associated signal in non-permeabilized HAEC using the Image J software, confirming that TRAP6, but not CXCL12, induces a substantial increase of membrane-associated VWF, as compared to control cells. Mean values ± SEM are displayed. *$p<0.05$; NS, not significant.

Supplemental Figure 3. Binding of soluble endoglin to αIIbβ3 integrin-expressing cells and platelets. **A**. Confocal microscopy analysis of sEng bound to CHO-αIIbβ3 cells. Monolayers of CHO cells were incubated with PE-Sol.Eng (red fluorescence) and with the anti-β3 integrin subunit (β3-Itg) antibody H1AG11 (green fluorescence). Nuclei were stained with DAPI (blue fluorescence). Parental CHO and CHO-αIIbβ3-WT cells were stimulated with PMA, as indicated. Samples were visualized by fluorescence confocal microscopy (Leica TCS-SP2-AOBS). White arrows indicate the presence of red dots corresponding to cell membrane-associated Sol.Eng. A representative experiment of three different ones is illustrated. **B.** Flow cytometry analysis of sEng binding to CHO-αIIbβ3 cells. Cells were detached from plates, stimulated or not with PMA, and then incubated with PE-sEng without or with a 10-fold excess of unlabeled sEng (last two columns on the right). Cell-bound PE fluorescence was analyzed by flow cytometry. A representative experiment is illustrated. **C.** Flow cytometry analysis of sEng binding to platelets stimulated or not with PMA. Platelets were incubated with unlabeled sEng followed by incubation with Alexa Fluor 488 anti-endoglin antibody. A488 fluorescence in non-aggregated platelets was analyzed by flow cytometry. The percentage of positive cells is indicated. A representative experiment is illustrated.

Videos 1-3. Three dimensional reconstruction of adherent platelets stained with phalloidin. Platelets were incubated for 30 min in wells coated with BSA (Video #1), fibrinogen (Video #2) or endoglin (Video #3). After washing the plates, platelets were labeled with phalloidin (red fluorescence), as described in Figure 1A. Samples were analyzed using fluorescence confocal microscopy (SP2, Leica). The representative videos show a 3D reconstruction made using LAS AF Lite software (Leica).

Videos 4-6. Effect of endoglin coating in flow adhesion assays. Platelet adhesion under flow was assessed by microscopy, using the microfluidic system of BioFlux (10x magnification), as described

in Fig. 1B. Calcein-labeled platelets were perfused in flow chambers coated with BSA (Video #4), fibrinogen (Video #5) or endoglin (Video #6), allowed to adhere in the absence of flow for 10 min, and then subjected to 2 dynes/cm^2 for 2 min. The flow goes from left to right, as indicated by the movement of the detached platelets. Platelets bound to the substrate were visualized by microscopy. Representative videos are included. Quantification of platelets that remained bound at the end of the experiment is represented in Fig. 1D.

Videos 7-8. Effect of CXCL12 on platelet flow adhesion to HUVEC. Calcein-labeled platelets were perfused in flow chambers coated with HUVEC in the absence (Video #7) or presence of CXCL12 (Video #8), allowed to adhere in the absence of flow for 10 min, and then subjected to 2 dynes/cm^2 for 2 min. Platelet adhesion under flow was assessed by microscopy, using the microfluidic system of BioFlux (10x magnification), as described in Supplemental Fig. 1C. The flow goes from left to right, as indicated by the movement of the detached platelets. Representative videos are provided. Quantification of platelets that remained bound at the end of the experiment is represented in Supplemental Fig. 1D.

Videos 9-12. Effect of endoglin silencing on platelet flow adhesion to HAEC. Calcein-labeled platelets were perfused in flow chambers coated with HAEC, previously untreated or treated with a siRNA specific for endoglin in the presence of CXCL12, as indicated. Rescue experiments of endoglin expression were performed by nucleofection of endoglin-silenced HAEC with the endoglin expression vector pCEXV-EndoL. Platelets were allowed to adhere in the absence of flow for 10 min, and then subjected to 2 dynes/cm^2 for 5 min. Platelet adhesion under flow was assessed by microscopy, using the microfluidic system of Maastricht Instrumentation and Replay software (Microvision Instruments, Evry, France) (10x magnification), as described in Fig. 3C. The flow goes from left to right, as indicated by the movement of the detached platelets. Quantification of platelets that remained bound at the end of the experiment is represented in Fig. 3C. Representative videos are provided. Video #9, HAEC; Video #10, HAEC + CXCL12; Video #11, HAEC + Endoglin-siRNA + CXCL12; Video #12, HAEC + Endoglin-siRNA +CXCL12 + pCEXV-EndoL.

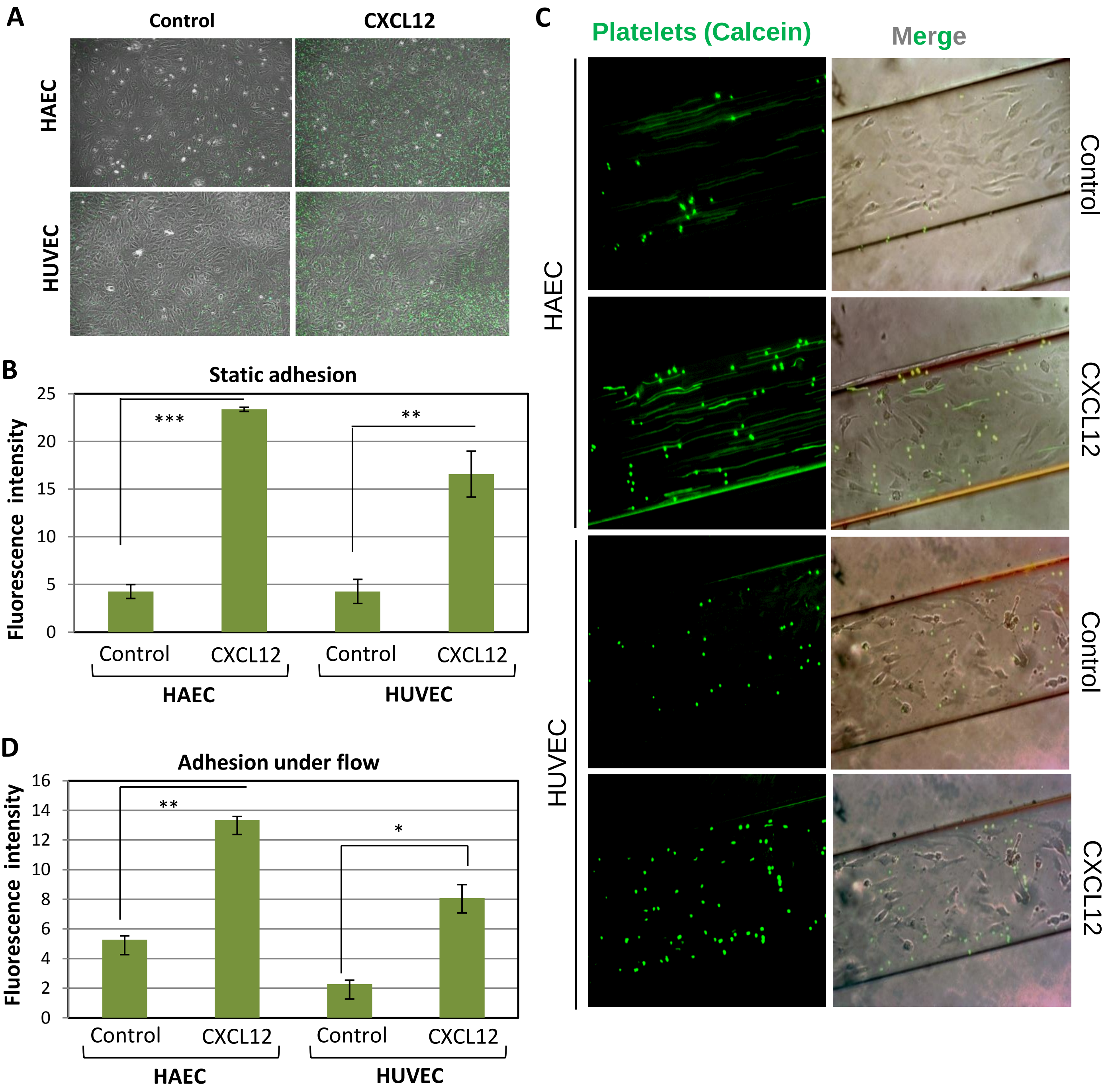

Supplemental Figure 1

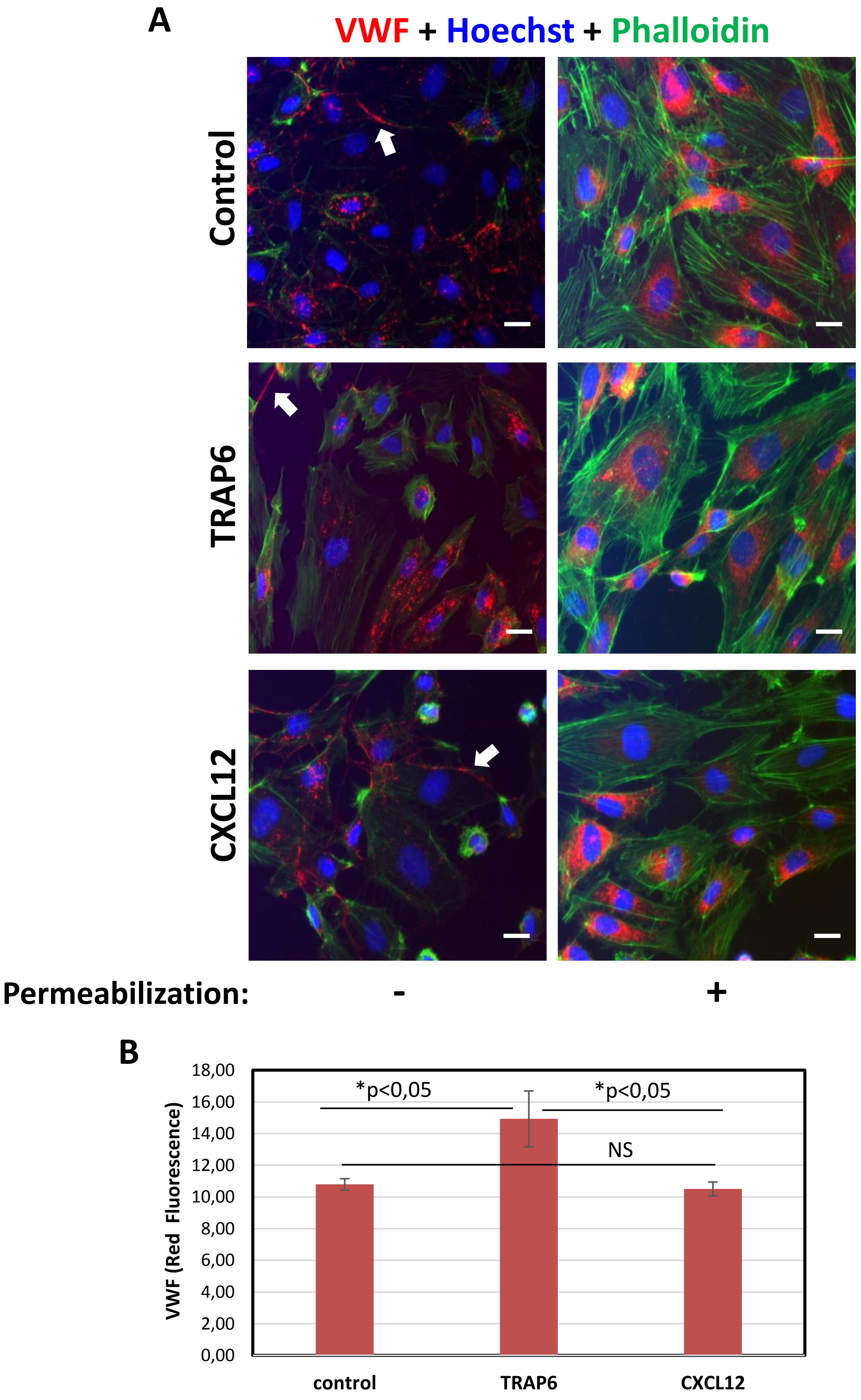

Supplemental Figure 2

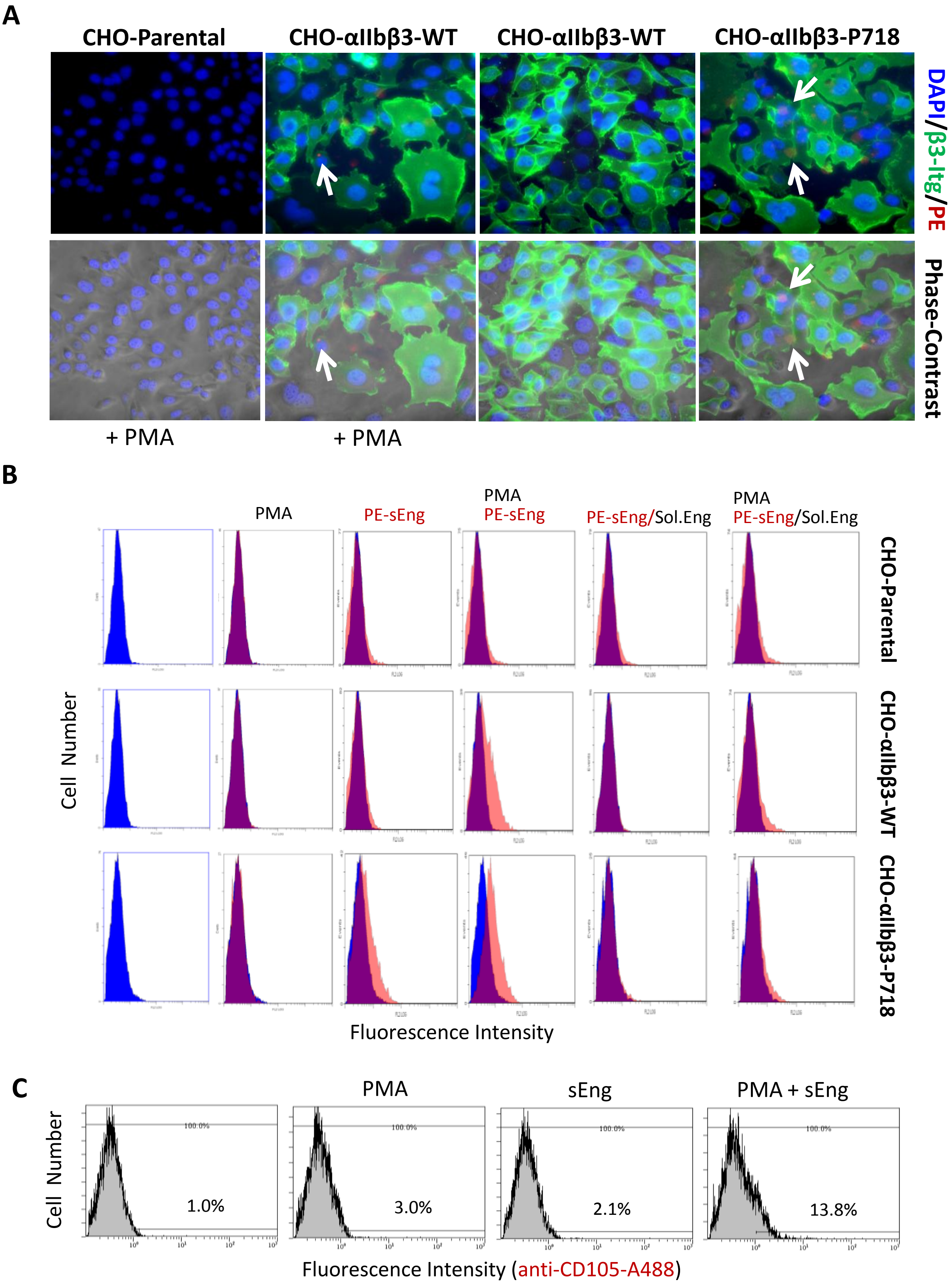

Supplemental Figure 3
